# Repeated human deciduous tooth-derived dental pulp cell reprogramming factor transfection yields multipotent intermediate cells with enhanced iPS cell formation capability

**DOI:** 10.1038/s41598-018-37291-2

**Published:** 2019-02-06

**Authors:** Miki Soda, Issei Saitoh, Tomoya Murakami, Emi Inada, Yoko Iwase, Hirofumi Noguchi, Shinji Shibasaki, Mie Kurosawa, Tadashi Sawami, Miho Terunuma, Naoko Kubota, Yutaka Terao, Hayato Ohshima, Haruaki Hayasaki, Masahiro Sato

**Affiliations:** 1 Division of Pediatric Dentistry, Graduate School of Medical and Dental Science, Niigata University, Niigata, Japan; 2 Department of Pediatric Dentistry, Kagoshima University Graduate School of Medical and Dental Sciences, Kagoshima, Japan; 3 Department of Regenerative Medicine, Graduate School of Medicine, University of the Ryukyus, Okinawa, Japan; 4 Faculty of Dentistry, Niigata University, Niigata, Japan; 5 Department of Oral Biochemistry, Graduate School of Medical and Dental Sciences, Niigata University, Niigata, Japan; 6 Division of Microbiology and Infectious Diseases, Graduate School of Medical and Dental Sciences, Niigata University, Niigata, Japan; 7 Division of Anatomy and Biology of the Hard Tissue, Graduate School of Medical and Dental Sciences, Niigata University, Niigata, Japan; 8 Section of Gene Expression Regulation, Frontier Science Research Center, Kagoshima University, Kagoshima, Japan

## Abstract

Human tissue-specific stem cells (hTSCs), found throughout the body, can differentiate into several lineages under appropriate conditions *in vitro* and *in vivo*. By transfecting terminally differentiated cells with reprogramming factors, we previously produced induced TSCs from the pancreas and hepatocytes that exhibit additional properties than iPSCs, as exemplified by very low tumour formation after xenogenic transplantation. We hypothesised that hTSCs, being partially reprogrammed in a state just prior to iPSC transition, could be isolated from any terminally differentiated cell type through transient reprogramming factor overexpression. Cytochemical staining of human deciduous tooth-derived dental pulp cells (HDDPCs) and human skin-derived fibroblasts following transfection with Yamanaka’s factors demonstrated increased ALP activity, a stem cell marker, three weeks after transfection albeit in a small percentage of clones. Repeated transfections (≤3) led to more efficient iPSC generation, with HDDPCs exhibiting greater multipotentiality at two weeks post-transfection than the parental intact HDDPCs. These results indicated the utility of iPSC technology to isolate TSCs from HDDPCs and fibroblasts. Generally, a step-wise loss of pluripotential phenotypes in ESCs/iPSCs occurs during their differentiation process. Our present findings suggest that the reverse phenomenon can also occur upon repeated introduction of reprogramming factors into differentiated cells such as HDDPCs and fibroblasts.

## Introduction

A variety of mammalian cells, including patient cells, have been successfully reprogrammed to generate induced pluripotent stem cells (iPSCs) through the enforced expression of stem cell-specific transcription factors such as POUF5F1 (OCT3/4 or OCT4; hereafter called OCT3/4), KLF4, SOX2, and c-MYC (OKSM)^1,2^. However, the efficiency of iPSC induction varies in different research laboratories owing to differences in the induction methods and starting materials employed. In particular, a low process efficiency often arises when primarily cultured cells are used as starting cells for reprogramming, as they contain both fully differentiated cells and heterogeneous intermediate populations, as exemplified by stem cells.

Human deciduous teeth from dental pulp cells (HDDPCs) are composed of several cell types including neuronal cells, fibroblasts, and undifferentiated mesenchymal cells. HDDPCs are useful as cellular resources because the deciduous teeth can be obtained from almost all children without pain. Moreover, we found that isolated HDDPCs of children can be easily propagated *in vitro* in preliminary studies because of their high proliferative potential and their clinical applicability in tooth regenerative medicine. More importantly, Tamaoki *et al*. suggested that iPSCs can be successfully obtained efficiently from dental stem cells^3^. Accordingly, in our previous study, we found that the stemness property in primarily isolated HDDPCs differed among individuals^4^. For example, two of five lines established exhibited increased expression of both alkaline phosphatase (ALP) and OCT3/4, known as stem cell markers; moreover, these two lines were more readily reprogrammed to generate iPSCs following the forced expression of reprogramming factors than the other remaining lines^4^. We inferred that cells enriched with immature stem cells may be susceptible to direct reprogramming. Furthermore, Yan *et al*. reported that when cells of dental tissue origin were infected with virus followed by a second infection 24 h later, the double infection by reprograming factors was more efficient than single infection^5^. These approaches appear to be useful for the enrichment of stem-like cells from differentiated cells lacking expression of pluripotency-related genes such as ALP and OCT3/4. Generally, a step-wise loss of pluripotential phenotypes in embryonic stem cells (ESCs)/iPSCs occurs during their differentiation process. For example, during differentiation of an ESC towards a true beta-cell phenotype, several selection steps are required such as embryoid body (EB) formation and subsequent formation of definitive endoderm, which is defined using definitive endoderm markers at various stages^6^. Similarly, during reprogramming of terminally differentiated cells to iPSCs the reverse phenomenon, considered a step-wise acquirement of pluripotential phenotypes, appears to occur. Notably, the Jaenisch laboratory demonstrated that ALP-positive cells first appear from 3 days after transfection of fibroblasts with reprogramming factors with OCT3/4 positive cells being present from 15 days prior to the appearance of distinct iPSC colonies. David and Polo (2016) showed that mesenchymal markers such as Thy and CD44 are lost whereas pluripotency makers such as ALP or SSEA-4 are gained during the period termed the ‘initiation phase’, a stage correlated with changes in morphology, such that fibroblast cells undergo a mesenchymal-to-epithelial transition^7^. This suggests that intermediate populations, as exemplified by ALP-positive cells, should exist during this de-differentiation process. Consistent with this, we have previously successfully isolated stem cell-like intermediate populations through the transfection of hepatocytes and pancreatic cells with reprogramming factors, and termed these isolated cell lines ‘induced tissue-specific stem cells (iTSCs)^8,9^. Furthermore, these isolated iTSCs exhibited no teratoma formation when they were subcutaneously grafted into immunodeficient mice^8,9^, suggesting the usefulness of these cells for tissue regeneration study. In the present study, we examined whether human iTSCs (hiTSCs) from HDDPCs (considered to show very low levels of expression of stem cell-related molecules) could be efficiently enriched by the employment of repeated transfection approaches to show stem cell-like properties such as mutlipotentiality and non-tumorigenicity.

## Results

### Generation of HDDPC-derived iPSCs

**Figure 1:**
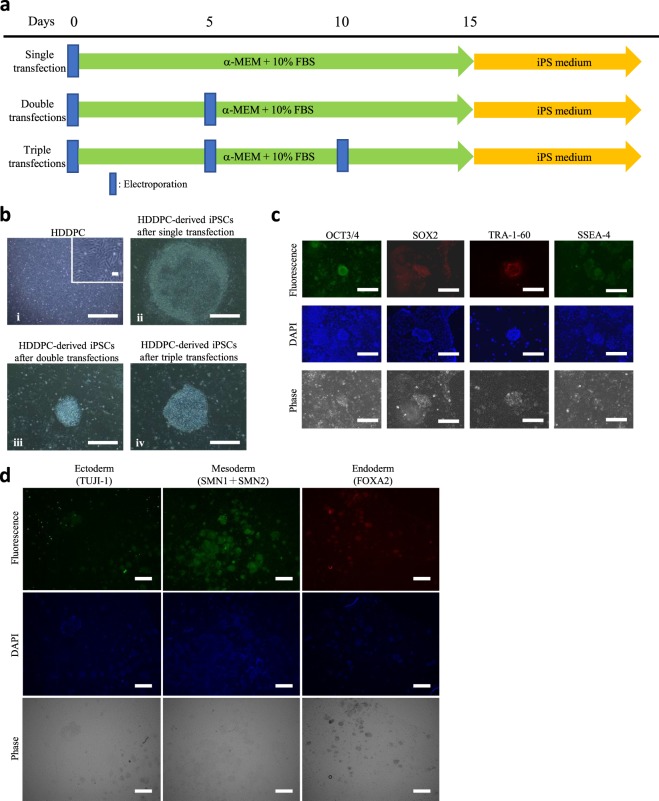
Generation of HDDPC-derived iPSCs. (**a**) Time-line for isolation of iPSCs from HDDPCs via single, double, or triple transfections. (**b**) Morphology of the HDDPCs (**a**) and HDDPC-derived iPSC colonies (**b**–**d**). iPSC colonies obtained after single (i), double (ii), or triple transfections (iii) from the HDDPC lines P02, P03, and P04, respectively. The magnified image is shown in the upper right portion of (i). Bar = 500 μm. (**c**) Immunocytochemistry of HDDPC (P01)-derived iPSC colonies using antibodies for OCT3/4, SOX2, TRA-1–60, and SSEA-4. Cells were stained by DAPI to visualise the location of nuclei. Bar = 500 μm. (**d**) Immunocytochemistry of embryoid body-derived outgrowth using antibodies for TUJI-1 (ectodermal marker), SMN1 + SMN2 (mesodermal marker), and FOXA2 (endodermal marker). Cells were stained by DAPI to visualise the location of nuclei. Bar = 500 μm.

We successfully obtained 6 HDDPC lines (termed ‘P01–P06’) from the patients, which were all found to exhibit low staining when assessed by histochemical analysis for ALP activity, except P01 (Table 1). We first examined whether HDDPCs could be reprogrammed to form iPSCs after single transfection with Yamanaka’s four reprogramming factors. The time-line for iPSC generation is shown in Fig. 1a. After single transfection of HDDPCs by electroporation, they were maintained in α-modified minimum essential medium (MEMα)/20% foetal bovine serum (FBS) for 15 days and thereafter in iPS medium for more 15 days. Of 6 HDDPC lines tested, only one (P01) exhibited distinct iPSC colonies at about 20 days after transfection (Fig. 1b-i,ii; Table 1). Immunohistochemical staining of these established iPSC colonies with antibodies demonstrated that they expressed stem cell markers such as OCT3/4, SOX2, TRA-1–60, and SSEA-4 (Fig. 1c). Furthermore, when outgrowth was allowed in the tissue culture dish from P01-derived EBs, the spread cells from the EBs exhibited various types of differentiated cells comprised of three germ layers (endoderm, mesoderm, and ectoderm) (Fig. 1d). However, the other 5 lines (P02–P06) retained a similar morphology as their parental cells even after 30 days following the first transfection.

**Table 1:** Summary of iPSC induction^a^ from 6 HDDPC lines through single or repeated (double or triple) transfections of vectors carrying reprogramming factors.

No. of lines	ALP activity^b^	Number of electroporations	Appearance of iPSC-like colonies^c^
P01	++	Single	+
P02	−	Single	−
P03	−	Single	−
P04	+	Single	−
P05	−	Single	−
P06	+	Single	−
P02	−	Double	+
P03	−	Double	−
P04	+	Double	+
P05	−	Double	−
P06	+	Double	+
P05	−	Triple	+

^a^HDDPCs were co-transfected with reprogramming vectors (pCE-OCT3/4, pCE-SK, and pCE-MYC) once, twice, or three times, according to the time-line described in Fig. 1a. After transfection, cells were first cultured in α-MEM + 10% FBS for 15 days and then in iPS cell medium containing β-FGF and 5 factors (PD98059, PD032590, CHIR99021, forskolin, and hLIF), except for P01 in which iPSC medium containing β-FGF and 4 factors (PD98059, PD032590, CHIR99021, and forskolin) was employed.

^b^ALP activity was evaluated by cytochemical staining for ALP activity using fixed HDDPCs. ++, >40% of cells exhibiting strong ALP activity; +, ~10% of cells exhibiting strong or moderate ALP activity; −, no cells showing ALP activity.

^c^The presence/absence of iPSC colonies 24 days after final transfection was judged as positive (+) or negative (−). If at least one colony per well was recognised, the well was judged as +.

We considered that the failure in acquiring iPSCs from the remaining 5 HDDPCs was because they had not yet reached the condition at which they could be readily reprogrammed to form iPSCs after transfection with reprogramming factors. Notably, Yan *et al*. demonstrated that repeated transfection with the reprogramming factors could increase the efficiency of iPSC generation^4^. We therefore tested this possibility by assessing whether the 5 lines (P02–P06) that were refractory to iPSC generation after single transfection could be successfully converted to form iPSCs upon repeated transfection with the reprogramming factors. For this purpose, cells were first transfected with Yamanaka’s four reprogramming factors, then at 5 days after transfection were harvested and subjected to the 2nd round of transfection using the same factors. These doubly-treated cells were maintained in MEMα/20% FBS for 5 days and then in iPS medium for another 15 days (Fig. 1a). Of the five HDDPC lines tested, three (P02, P04, and P06) exhibited formation of iPSC-like colonies approximately 20 days after the 2nd-round transfection (Fig. 1b–iii; Table 1). The other lines (P03 and P05) retained a form of fibroblastic morphology even after 30 days after the 2nd transfection. For exploring the possible conversion of these cells to iPSCs, a 3rd round of transfection was performed using the P05 line, as shown in the time-line shown in Fig. 1a. Approximately 20 days after the 3rd transfection, P05 successfully exhibited formation of iPSC-like colonies (Fig. 1b-iv; Table 1).

### Generation of intermediate stem-like cells from primary HDDPCs

From the results of the previous experiment, it was shown that the HDDPCs that failed to convert to iPSCs after the single transfection with Yamanaka’s four reprogramming factors could be successfully reprogrammed to form iPSCs when subjected to repeated transfections. These findings suggest that HDDPCs (which are refractory to iPSC generation after single transfection) come to reach the condition at which cells are easily reprogrammed to iPSCs after repeated transfection. In other words, they are in an intermediate state between fully differentiated cells such as parental HDDPCs and iPSCs. Notably, ALP expression is closely associated with undifferentiated immature cells including somatic stem cells and ESCs/iPSCs^4,10^. Furthermore, after transfection with the reprogramming factors, ALP-positive cells first appear prior to the formation of OCT3/4 positive iPSC-like colonies^10^. This suggests the potential of ALP as a useful marker for defining cells at an intermediate state.

**Figure 2:**
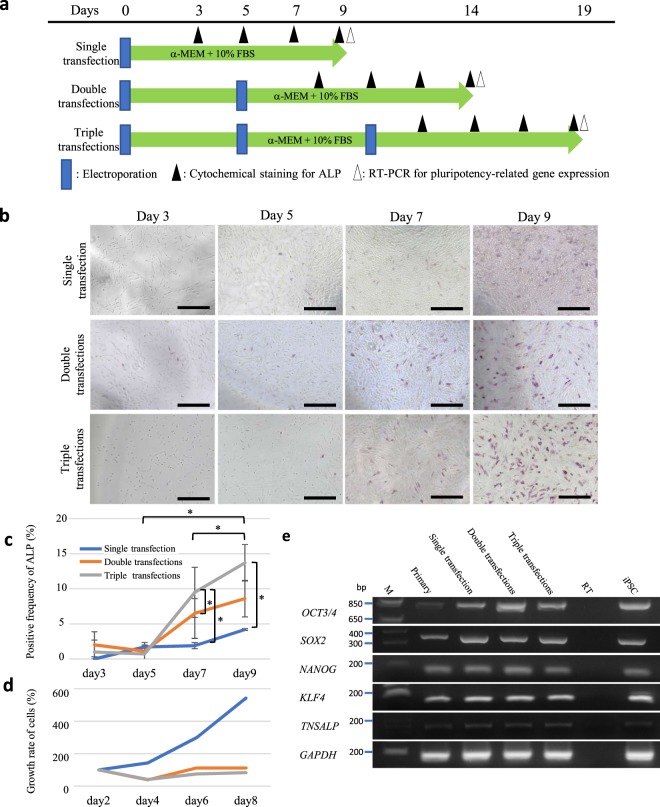
Increased pluripotency-related gene expression in HDDPCs after repeated transfection with Yamanaka’s four reprogramming factors. (**a**) Time-line for assessment of pluripotency-related gene expression in HDDPCs after repeated transfection. (**b**) Cytochemical evaluation of ALP activity in the HDDPCs after repeated transfections with the reprogramming factors. HDDPCs (P05 line) were transfected with Yamanaka’s four reprogramming factors once, twice, or three times. The treated cells were subjected to cytochemical staining for ALP activity at 3, 5, 7, and 9 days after the final transfection, as shown in (**a**). Bar = 500 μm. (**c**,**d**) Ratio of ALP-positive cells (**c**) and proliferation curves (**d**) for HDDPCs when the P05 line was subjected to transfection once, twice, or three times. After the final transfection, cells were seeded onto 4- or 24-well plate, and fixed (for cytochemical staining for ALP activity) or harvested (for counting the cell number) at the days indicated. (**e**) RT-PCR analysis of HDDPCs after the repeated transfections. HDDPCs (P05 line) were transfected with Yamanaka’s four reprogramming factors once, twice, or three times, and harvested 9 days after the final transfection for RT-PCR analysis for detection of mRNA expression of OCT3/4, SOX2, NANOG, and KLF4, tissue-nonspecific alkaline phosphatase (TNSALP), and glyceraldehyde-3-phosphate dehydrogenase (GAPDH) (as control). Lane 1, HDDPCs (P05 line); lane 2, HDDPCs after the single transfection; lane 3, HDDPCs after the double transfection; lane 4, HDDPCs after the triple transfection; lane 5, no template (as negative control); lane 6, iPSCs (as positive control). M, 100-bp ladder markers.

Therefore, we next examined the possible appearance of ALP-positive HDDPCs after transfection with Yamanaka’s four reprogramming factors. In this case, we employed the P02 line, which is negative for ALP activity and found to be refractory to iPSC formation after the 1st-round transfection with the reprogramming factors (Table 1). On 3, 5, 7, and 9 days after transfection, cells were fixed and subjected to cytochemical staining for ALP activity (Fig. 2a). Gradual increase in ALP activity was clearly discernible as days in culture proceeded (upper columns in Fig. 2b). For the 2nd- round transfection, cells at 5 days after the 1st transfection were harvested and immediately subjected to retransfection. On 3, 5, 7, and 9 days after the 2nd-round transfection, cells were fixed for the detection of ALP activity. As shown in the case of the 1st transfection, a gradual increase in ALP activity was also seen as days in culture proceeded (middle columns in Fig. 2b). This pattern was observed as well when triple transfections were performed using the same line (lower columns in Fig. 2b). The number of ALP-positive cells/well in the double and triple transfections groups was significantly higher than that in the single transfection group when cytochemical staining was performed on 7 and 9 days after transfection (Fig. 2c). Notably, the proliferation rate in the single transfection group was also higher than those in the double and triple transfection groups (Fig. 2d). These findings suggest a correlation between repeated transfections with the reprogramming factors and elevated numbers of ALP-positive cells.

To test whether cells growing after repeated transfections with the reprogramming factors might also express other pluripotency-rerated markers such as OCT3/4, SOX2, NANOG, KLF4, and TRANSP, reverse transcription-polymerase chain reaction (RT-PCR) analysis was carried out using the samples (P02) harvested on 9 days after single, double, or triple transfection. Increased expression of these pluripotency-rerated markers was already discernible in HDDPCs 9 days after the single transfection (Fig. 2e). The levels of these mRNAs appeared to be almost equivalent to that found in the established iPSCs^4,11^. Notably, OCT3/4 and TRANSP tended to show an increase in mRNA expression as the transfection was repeated. However, regardless of pluripotency-related gene expression, HDDPC morphology remained fibroblastic, similar to its parental cells, at 9 days after the 1st transfection. Therefore, we considered that these cells, termed as “hiTSC-D”, were in an intermediate state between HDDPCs and iPSCs.

### Generation of intermediate stem-like cells from human fibroblasts

**Figure 3:**
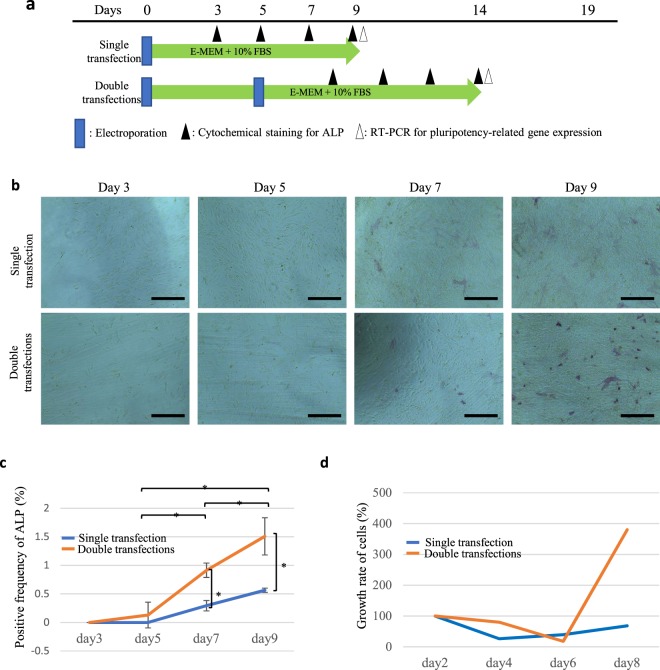
The changes of ALP activity in human skin-derived fibroblasts after repeated transfection. (**a**) Time-line for assessment of pluripotency-related gene expression in fibroblasts after repeated transfection. (**b**) Cytochemical evaluation of ALP activity in the fibroblasts after repeated transfection with the reprogramming factors. Fibroblasts were transfected with Yamanaka’s four reprogramming factors once or twice. The treated cells were subjected to cytochemical staining for ALP activity at 3, 5, 7, and 9 days after the final transfection, as shown in (**a**). Bar = 500 μm. (**c**) Ratio of ALP-positive cells and (**d**) proliferation curves for fibroblasts when the parental fibroblasts were subjected to transfection once or twice. After the final transfection, cells were seeded onto 4- or 24-well plate, and fixed (for cytochemical staining for ALP activity) or harvested (for counting the cell number) at the days indicated.

Similar to the protocol for HDDPCs, human skin-derived fibroblasts were fixed and subjected to cytochemical staining for ALP activity on days 3, 5, 7, and 9 after transfection (Fig. 3a). Gradual increase in ALP activity was also clearly discernible as days in culture proceeded (Fig. 3b). As shown in the case of the 1st transfection, a gradual increase in the frequency of ALP activity was also seen as days in culture proceeded (Fig. 3c). The number of ALP-positive cells/well, examined on days 7 and 9 after transfection, in the group subjected to double transfections was significantly higher than that in the group subjected to single transfection (Fig. 3c). Similar to hiTSC-D, we termed these ALP-positive human fibroblast-derived intermediate state cells as “hiTSC-F”.

### Microarray analysis compared with intermediate stem-like cells from HDDPCs and human fibroblasts

**Figure 4:**
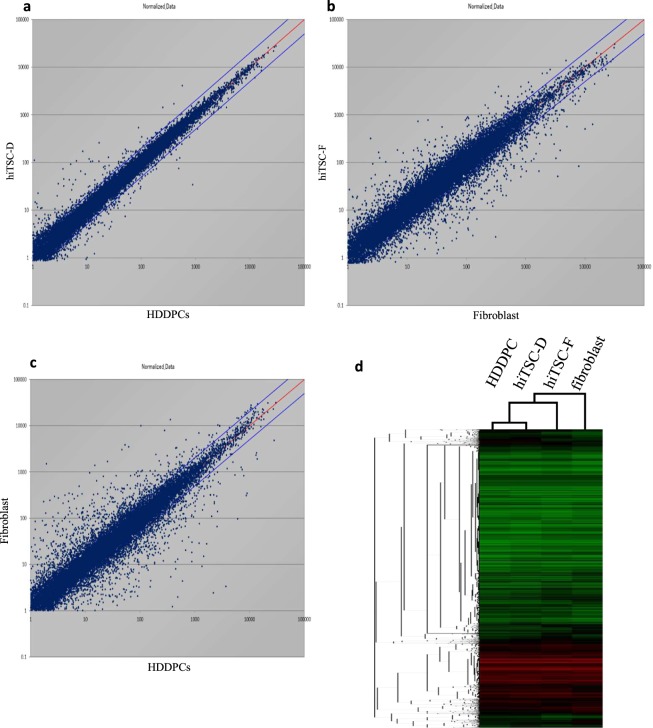
Microarray analysis. Transcriptome Analysis Console (TAC) Software from Affymetrix was used to analyse global gene expression patterns between HDDPCs and hiTSC-D (**a**), fibroblasts and hiTSC-F (**b**), and HDDPCs and fibroblasts (**c**). (**d**) Unsupervised hierarchical clustering of gene expression profiles of HDDPCs, hiTSC-D, fibroblasts, and hiTSC-F. Each column represents one biological sample.

We compared the global gene expression profiles of HDDPCs, human skin-derived fibroblasts, hiTSC-D (obtained 10 days after the single transfection), and hiTSC-F (obtained 10 days after the single transfection) using microarray analysis. Of the 24,462 genes examined, the distributions differed by >2-fold between HDDPCs and hiTSC-D, human fibroblasts and hiTSC-F, and HDDPCs and human fibroblasts as shown in Fig. 4 and Supplemental Dataset. These data indicate a close similarity in gene expression profile between HDDPCs and hiTSC-D (Fig. 4a), which is in contrast to that between skin-derived fibroblasts and hiTSC-F (Fig. 4b) or between HDDPCs and skin-derived fibroblasts (Fig. 4c). Unsupervised hierarchical clustering of the gene expression profiles of these cells also confirmed these notions: namely, hiTSC-D clustered more closely with HDDPCs than with fibroblasts (Fig. 4d).

### Multi-differentiation potency of intermediate cells from HDDPCs

**Figure 5:**
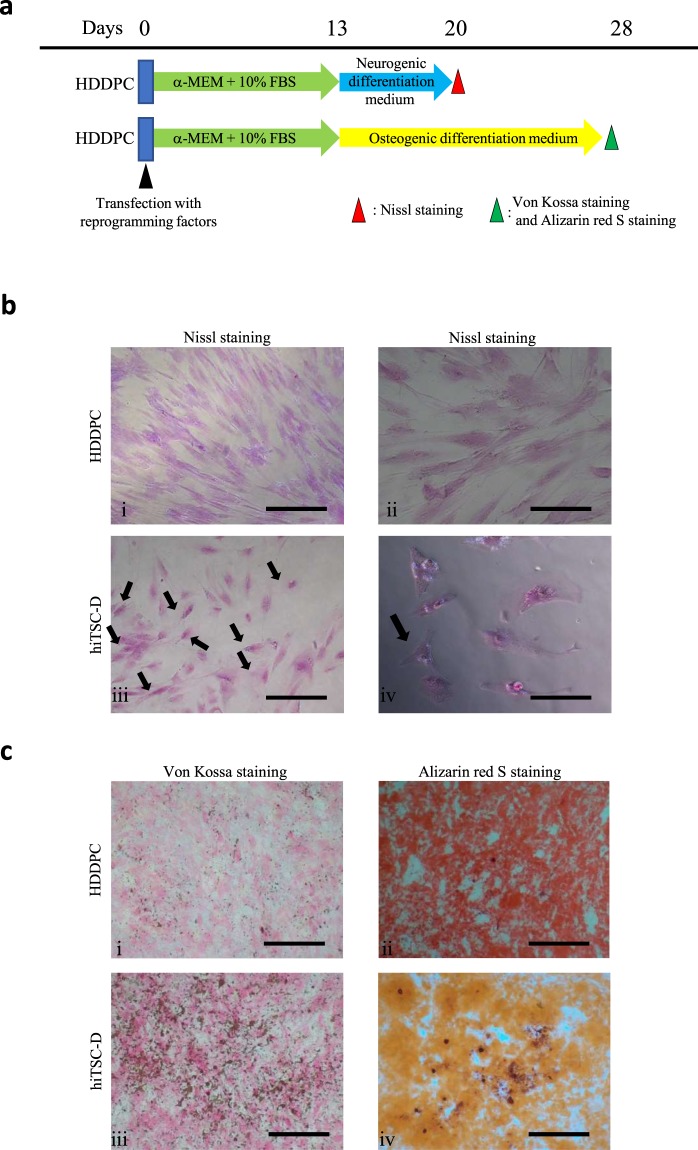
Differentiation induction of the intermediate HDDPCs. (**a**) Time-line for differentiation induction of the HDDPC line P05 (13 days after the single transfection with Yamanaka’s four reprogramming factors) into neurogenic or osteoblastic lineage. (**b**) Neuronal cell induction 7 days after incubation in neurogenic medium. Nissl staining demonstrated that Nissl bodies are clearly discernible around the nucleus in the transfected cells (arrowed) in (iii), whereas no such bodies are discernible in the untransfected cells in (i). Bar = 500 μm (i) and (iii), 50 μm (ii) and (iv). (**c**) Osteoblastic cell induction 15 days after incubation in osteogenic medium. Von Kossa staining in (i) and (iii), and staining with Alizarin red-S in (ii) and (iv). Bar = 500 μm.

To test whether these intermediate cells possessed multi-differentiation potential, cells (P02) harvested 13 days after the 1st transfection were subjected to differentiation induction to osteoblastic or neurogenic lineage, as shown in Fig. 5a. Von Kossa staining revealed the presence of mineralisation (stained brown-black) in the cells treated with STK3™ (Fig. 5b-iii), whereas no deposition was observed in cells cultured in the normal medium (Fig. 5b-iv). Furthermore, the presence of calcific deposition by cells of an osteogenic lineage was detected when the cells were treated with osteogenic differentiation medium STK3™ for 7 days and then stained with Alizarin red S (Fig. 5c-ii). In contrast, however, in the control culture, no such alteration was observed (Fig. 5b-i). We also detected the presence of neuronal cells, as revealed by Nissl staining (Fig. 5b-iii; arrows in Fig. 5b-iv) in the P02 cells cultured for 7 days in STK3™ but not in the control culture (Fig. 5b-i,ii).

We next assessed the *in vivo* growth capacity of the P02-derived intermediate cells by xenogenic intrapancreatic parenchymal transplantation into nude mice, which allows the growth and differentiation of a small number of iPSCs as well as proliferative tumours such as the pancreatic cancer cell line SUIT-2 and murine teratocarcinoma F9 cells *in vivo*^12^. When the P02 cells at 13 days after reprogramming factor transfection were transplanted into the pancreas of four nude mice (approximately 10^6^ cells/site; a total of three sites injected), no visibly discernible tumours could be detected in the pancreas for four months (data not shown).

## Discussion

The development of iPSCs has shed light on the possibility of obtaining autologous pluripotent embryonic-like stem cells, circumventing the need for somatic cell nuclear transfer-treated embryos^2,13^. However, the reprogramming process appears highly inefficient and likely depends on many factors including the age, type, passage number, and origin of the primary cells used^14^. For example, our previous attempt to reprogram a total of six primary HDDPC lines by transfection with plasmids carrying Yamanaka’s four reprogramming factors, only two were successfully reprogrammed to form iPSCs whereas the other four lines were refractory to this treatment^4^. Moreover, we found a close association between a higher ALP activity of cells and the susceptibility to reprogramming to form iPSCs^4^. As ALP activity is closely associated with immature undifferentiated cells such as ESCs/iPSCs and stem cells^15^, we considered that HDDPCs showing strong ALP activity may be abundantly enriched with stem cells, which may in turn be more susceptible to form iPSCs after transfection with the reprogramming factors. In accordance with this notion, in the present study we found that only an ALP-positive HDDPC line (P01) that was newly established was successfully induced to form iPSCs upon initial transfection (Table 1). However, the other five lines (P02–06), which were judged as those with low or absent ALP activity, failed to form iPSCs (Table 1).

We then addressed whether it was possible to convert these five lines (P02–06) to form iPSCs? Based on their ALP-negative status, we considered that application of conditions to elevate ALP activity might constitute a plausible option. Following the results of Yan *et al*.^4^, we expected that repeated transfection with the reprogramming factors would result in generation of ALP-positive cells and enhanced potential for iPSC generation. Consistent with this, three of five lines tested exhibited successful generation of iPSCs (Fig. 1b-iii; Table 1). Furthermore, P05, a line that failed to convert into iPSCs even after the 2nd-round transfection, was successfully converted to form iPSCs after the 3rd-round transfection (Fig. 1b-iv; Table 1). These results led us to suppose that repeated transfection of HDDPCs might constitute a useful tool for the enrichment of ALP-positive stem cells that are more conducive to iPSC generation. Accordingly, when ALP activity in the ALP-negative P02 line was assessed cytochemically during the process of reprogramming, a gradual increase in ALP activity was observed (Fig. 2b; upper columns; Fig. 2c). Notably, this pattern was also detected when the P02 line was subjected to 2nd- and 3rd-round transfections (Fig. 2b; middle and lower columns; Fig. 2c). Furthermore, the frequency of ALP-positive cells increased as the number of transfections increased (Fig. 2c). Analysis of pluripotency-related gene expression by RT-PCR also demonstrated that the HDDPCs were in a stem-like cell state at 9 days after transfection with the reprogramming factors, which was very similar to that of iPSCs at the molecular level (Fig. 2e) although their morphology remained fibroblastic, which differed from that of iPSCs. Taken together, these findings indicate that repeated transfections are beneficial for converting ALP-negative cells to stem-like cells as well as for iPSC generation.

Notably, it was possible to obtain iPSCs after the 4^th^-round transfection with four reprogramming factors. However, 7 out of 10 lines tested failed to form iPSC-like colonies on passage 7 and afterward (Supplemental Fig. 1). Furthermore, it was reported that repeated transfection with reprogramming factors can lead to generation of iPSCs in naïve state (Supplemental Fig. 1). Unfortunately, we failed to detect such naïve state iPSCs after the 4^th^-round transfection.

Notably, ALP activity still appeared to be low in the cells at 3 days after the 3rd-round transfection (Fig. 2b lower column; Fig. 2c). However, these cells corresponded to the cells at 12 days after the 1st transfection (Fig. 2a), which already showed relatively strong ALP activity, similar to Day 9 cells (Fig. 2b, upper columns). This implies that the ALP expression observed in the Day 9 cells may be labile, with rapid loss of pluripotency-related gene expression potentially occurring immediately after re-seeding. Subsequent analyses should therefore assess whether the Day 9 cells immediately after re-seeding can continue to express pluripotency-related genes.

Similar to the case of transfection of HDDPCs using reprogramming factors, the rate of ALP-positive human skin-derived fibroblasts after the 2^nd^-round transfection was higher than that of cells obtained after the single transfection (Fig. 3c). The number of ALP-positive fibroblasts gradually increased after the first transfection. This tendency was also seen when the 2^nd^-round transfection was performed: the number of ALP-positive cells appeared to peak at 7 and 9 days after transfection (Fig. 3c). These findings suggest that fibroblasts can also be reprogrammed partially, and similar to the case of HDDPCs, ALP activity was also a useful marker for cells at early stage of de-differentiation.

Cell proliferation after transfection with reprogramming factors appeared to differ between HDDPCs and fibroblasts. For example, HDDPCs exhibited faster cell proliferation after the 1^st^ transfection but not after the 2^nd^ and 3^rd^-round transfection (Fig. 2d). On the other hand, fibroblasts exhibited faster cell proliferation than their untransfected parental cells on day 8 and afterward after the 2^nd^-round transfection (Fig. 3d). It remains unknown why the mode of cell proliferation differs between these two cells, but as shown in the results of microarray analysis, it may reflect the difference in gene expression profile between them.

We performed microarray analysis for possible differences in gene expression profile between HDDPCs and hiTSC-D, fibroblasts and hiTSC-F, or HDDPCs and fibroblasts. This cluster analysis demonstrated that HDDPCs and hiTS-D cells had the most similar molecular signatures among these 4 cells (see Fig. 4a,d). We have already indicated that HDDPCs are thought to contain a number of stem cells as they exhibited ALP activity and were enriched with transcripts derived from stemness factors such as OCT3/4 and SOX2^4^. This is in contrast to the case of fibroblasts, which are thought to have less amounts of stemness factors. Gene expression profile between fibroblasts and hiTSC-F appears to differ (see Fig. 4b,d), suggesting alteration in the mode of gene expression in fibroblasts after transfection with reprogramming factors. Furthermore, gene expression profile between HDDPCs and fibroblasts differed (see Fig. 4c,d), suggesting that both types of cells differ at the gene expression level.

**Figure 6:**
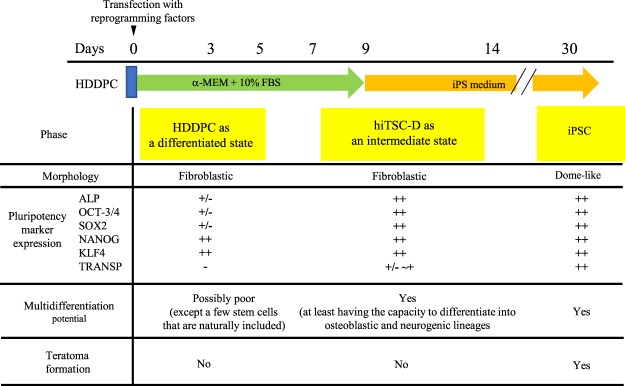
Summary of the properties of intermediate state cells (hiTSC-D) generated around 9 days after transfection with Yamanaka’s four reprogramming factors.

Since different molecular transitions during reprogramming were first documented by the laboratories of Jaenisch and Hochedlinger in 2008^16,17^, the reprogramming process has been roughly grouped into three phases (i.e., initiation, maturation, and stabilisation) by Samavarchi-Tehrani *et al*.^18^. In the initiation phase, fibroblast-specific surface markers such as THY1 and CD44 are down-regulated, whereas the pluripotency markers ALP followed by SSEA1 are gained, along with reactivation of telomerase activity^16^. This process is also characterised by a loss of the somatic cell signature, such as decreased expression of transcription factors SNAIL1/2 and ZEB1/2^17,19,20^ and the gain of an epithelial-associated miRNA-200 family^18,21^. Polo *et al*. also noted that ALP and FBXO15, early markers of pluripotent cells, gradually increased their expression, whereas endogenous transcripts of OCT3/4 and SOX2 were detectable only late during iPSC generation^10^. Furthermore, single-cell analysis of 26 genes as well as fluorescence-activated cell sorting analysis demonstrated that gene expression changes occur homogeneously at early (day 0–3) and late time points (day 9 onward), whereas they are heterogeneous at intermediate stages (day 6–9)^10^. In the present study, we showed an increased expression of ALP around 9 days after a single transfection (Fig. 2b, upper columns), which may correspond to the intermediate stages described by Polo *et al*.^10^. Taken together, it may be plausible to consider that the intermediate stage between HDDPCs and iPSCs should exist around 9 days after transfection, as depicted in Fig. 6. Cells at this stage are morphologically indistinguishable from their parental HDDPCs but express pluripotency-related markers such as ALP and OCT3/4, as do iPSCs. Such features resemble those of various types of stem cells (i.e., bone marrow-derived stem cells and mesenchymal stem cells), all of which exhibit *in vitro* multipotentiality and non-tumorigenicity when grafted into immune-deficient mice. Consistent with this, we observed that HDDPCs, at 13 days after singe transfection with the Yamanaka’s four reprogramming factors, had the ability to differentiate into at least two types of cells, osteoblastic and neuronal cells (Fig. 5b). Moreover, intrapancreatic parenchymal inoculation of these cells produced no visible teratomas in five nude mice observed over a course of four months (data not shown). Thus, these HDDPC-derived intermediate stage cells were considered as iTSCs and are termed ‘hiTSC-Ds (induced tissue-specific stem cells from deciduous tooth-derived dental pulp cells)’.

In conclusion, we successfully produced iPSCs from HDDPCs (judged as cells refractory to convert to iPSC formation by a single transfection) through repeated transfections with Yamanaka’s four reprogramming factors. During the reprogramming process, we revealed the presence of intermediate cells, termed hiTSC-Ds, having the stemness properties of molecular profile, multipotentiality, and non-tumorigenicity. These cells will likely have potential application towards the study of mammalian dental tissue regeneration.

## Methods

### Animals

All animal experiments were performed in agreement with Niigata University Committee on Recombinant DNA Security guidelines (permit no. SP00636 dated 1st Aug. 2016) and with Animal Care and Experimentation Committee of Niigata University approval (permit no. 28 No163-1 dated 24th Jun. 2016) according to the “Guide for the Care and Use of Laboratory Animals” of the National Academy of Sciences, USA. All surgeries were performed under three anaesthetics (medetomidine, midazolam, and butorphanol)^22^, and all efforts were made to minimise suffering. For intrapancreatic tumour cell inoculation, eight- to twenty-week-old immunodeficient female mice (Balb/c-nu/nu, CLEA Japan, Tokyo, Japan) were used.

### Primary cell culture of HDPPCs and human fibroblasts

HDDPCs were collected from patients after obtaining written informed consent from their legal guardians; study protocols were conducted in accordance with the tenets of the Declaration of Helsinki and approved by the Ethical Committee for Use and Experimentation of the Niigata University Graduate School of Medical and Dental Sciences (permit no. 28-R21-6-20 dated 21st Nov. 2016). HDDPCs were collected from patients after obtaining informed consent from their legal guardians; study protocols were approved by the Ethical Committee for Use and Experimentation of the Niigata University Graduate School of Medical and Dental Sciences (permit no. 28-R21-6-20 dated 21^st^ Nov. 2016). HDDPCs were isolated as described previously^23^, with slight modifications^4^. Briefly, pulp tissue was removed from the deciduous teeth of four young patients and digested with a solution of 3 mg/mL collagenase type I (#17100-017; Invitrogen, Carlsbad, CA, USA) and 4 mg/mL dispase (#410810077, Roche Applied Science, Basel, Switzerland) in Dulbecco’s phosphate-buffered saline (DPBS) (#D8537; Sigma-Aldrich Co., Dorset, UK) for 25 min at 37 °C. Isolated pulp cells were cultured in MEMα (#135–15175, Wako Pure Chemical Industries, Ltd., Osaka, Japan) with 20% foetal bovine serum (FBS), 100 μM L-ascorbic acid-2-phosphate (#323–44822; Wako), 50 U/mL penicillin, and 50 mg/mL streptomycin (herein referred to as ‘MEMα/20% FBS’) at 37 °C in 5% CO_2_. After 3–7 passages, HDDPCs were used for transfection experiments.

For comparison, normal skin-derived human fibroblast (#JCRB0075; Japanese Collection of Research Bioresources, Ibaraki, Japan) was cultured in Dulbecco’s modified Eagle’s medium (DMEM) (#11995040; Thermo Fisher Scientific K.K. Tokyo, Japan) with 10% FBS, 50 U/mL penicillin, and 50 mg/mL streptomycin at 37 °C in 5% CO_2,_ and used for transfection experiments after 3–5 passages.

### Generation of HDDPC-derived iPSCs

HDDPC-derived iPSCs were generated using our own protocol^11^ with slight modifications^4^. Briefly, HDDPCs (approximately 1 × 10^5^) were transfected with three kinds of plasmids [2 μg each: pCXLE-hOCT3/4-shp53 (carrying human *OCT3/4* cDNA and shRNA for human p53), pCXLE-hUL (carrying human L-*MYC* and *LIN28* cDNAs), and pCXLE-hSK (carrying human *SOX2* and *KLF4* cDNAs); purchased from Addgene (Cambridge, MA, USA)], using an electroporation-based Neon microporation system (#MPK5000; Invitrogen) in 100 μL volume. Transfected cells were seeded in a gelatin-coated 6-well plate (#4810-020; Iwaki Glass Co., Tokyo, Japan) containing MEMα/20% FBS. After 15 days, cells were trypsinised and re-seeded onto mytomycin C (MMC)-treated (#M4287; Sigma-Aldrich) mouse embryonic feeder cells in a 60-mm gelatin-coated dish (#4010-010; Iwaki Glass), with human ESC culture medium iPSellon (#007001; Cardio, Kobe, Japan) supplemented with 5 ng/mL recombinant human basic fibroblast growth factor (#064-04541; Wako) (herein referred to as ‘iPS medium’). For repeated transfections (double transfection), cells were harvested at 5 days after the 1st transfection, subjected to the 2nd transfection with reprogramming factors, using the same conditions, seeded onto a gelatin-coated 6-well plate containing MEMα/20% FBS, cultured for 10 days (Fig. 1a), then cultured in iPS medium for an additional 15 days. For triple transfection, cells were harvested 5 days after the 2nd transfection, transfected with the reprogramming factors as described in Fig. 1a, seeded onto a gelatin-coated 6-well plate containing MEMα/20% FBS, cultured for 5 days (Fig. 1a), then cultured in iPS medium for 10 additional days.

The resulting iPS colonies were immunocytochemically stained using antibodies as described below, or further propagated on MMC-treated mouse embryonic feeder cells for preparing cell stocks and karyotypic analysis.

### Generation of intermediate stem-like cells from primary HDDPCs

HDDPCs (approximately 1 × 10^5^) judged as having no ALP activity underwent the 1st transfection then were seeded onto a gelatin-coated 24-well plate (#4820-020, Iwaki Glass) at approximately 1 × 10^4^ cells/well to check ALP and proliferative activity (Fig. 2a). Cells were cultured in MEMα/20% FBS throughout the experiment. Cells were fixed with 4% paraformaldehyde (PFA) (for checking ALP activity) or harvested at 3, 5, 7, and 9 days after seeding by trypsinisation (for checking proliferative activity). For repeated transfections (double transfection), at 5 days after the 1st transfection, cells were harvested by trypsinisation for the 2nd transfection with reprogramming factors. The treated cells were seeded onto a gelatin-coated 24-well plate for checking ALP and proliferative activity on the days indicated after seeding, as shown in Fig. 2a. For triple transfection, at 5 days after the 2nd transfection, cells were harvested for the 3rd transfection, as shown in Fig. 2a. After final seeding, cells were examined for ALP and cell proliferative activities as indicated. In some cases, cells were harvested at 19, 14, and 9 days after the final transfection for single, double, or triple transfection, respectively (Fig. 2a), for mRNA expression analysis using RT-PCR. HDDPCs 13 days post-1st transfection were also subjected to *in vitro* differentiation induction and *in vivo* teratoma formation, as shown below. Using the same procedure used for generation of HDDPC-derived hiTSCs, fibroblasts were transfected twice with the reprogramming factors and checked for ALP activity.

### Proliferative rate of HDDPCs and fibroblasts

To evaluate cell proliferation rates, cells (approximately 1 × 10^4^) seeded onto a gelatin-coated 24-well plate were collected by trypsinisation after 1, 3, 5, 7, or 9 days and the cell number counted using a disposable haemocytometer. At least 3 wells per line were examined and the average cell number was plotted. The data were expressed as the mean ± standard error. Two groups were compared using the Student *t*-test. The differences in each group were considered significant if *P* < 0.05. All statistical analysis methods were performed in accordance with the relevant guidelines and regulations.

### Immunocytochemical staining

Cells were fixed with 4% PFA for 15 min, treated with 0.1% Triton X-100 (#B161–0407; Bio-Rad Laboratories, Hercules CA, USA) in DPBS for 3 min for cell permeabilisation, and immersed in 20% AquaBlock (#PP82; EastCoast Bio, North Berwick, ME, USA) for 30 min, all at room temperature. Cells were stained with primary antibodies against OCT3/4 (#MAB4401; 1:400, Merck Millipore, Billerica, MA, USA), SOX2 (#SAB2701974; 1:100, Sigma-Aldrich), SSEA-4 (#MAB4304; 1:500, Merck Millipore), TRA-1–60 (#MAB4360; 1:500, Merck Millipore), TUJI-1 (#ab18207; 1:100, Abcam, Cambridge, England, UK), SMN1 + SMN2 (#ab124438; 1:100, Abcam), and FOXA2 (#ab5074; 1:250, Abcam) overnight at 4 °C. After washing with DPBS, cells were next reacted with the secondary antibodies, FITC-conjugated goat anti-mouse IgG γ chain antibody (#AP503F; 1:100, Merck Millipore), Alexa Fluor 594-conjugated goat anti-mouse IgM heavy chain (#A-21044; 1:100, Invitrogen), Alexa Fluor 594-conjugated goat anti-mouse IgG H&L (#ab150116 1:200, Abcam), Alexa Fluor 488-conjugated goat anti-rabbit IgG H&L (#ab150077 1:200, Abcam), or Alexa Fluor 488- conjugated donkey anti-goat IgG H&L (#ab150129; 1:200, Abcam) for approximately 2 h at 4 °C. Cells were washed with PBS, nuclear stained using 6-diamidino-2-phenylindole (DAPI) (#H-1200; Funakoshi, Tokyo, Japan) for 30 min at room temperature, then slide mounted; florescence was inspected and recorded using a fluorescence microscope.

### ALP assay

Cytochemical ALP activity assay was performed using 4% PFA-fixed cells with an Alkaline Phosphatase Staining Kit II (#00–0055; STEMGENT, Cambridge, MA, USA), which utilised α-naphtholum-coupled diazonium salt to stain ALP per manufacturer protocol. Cells in the centre well portion were photographed and the stained and unstained cells were manually counted. A total of approximately 1 × 10^5^ cells per well were counted. Three wells per transfection group were examined and the average cell number was plotted.

### RT-PCR analysis

Total RNA from each sample was isolated using the RNA Mini Kit (#50204; Qiagen, Tokyo, Japan) as previously described in Murakami *et al*.^24^. A negative, no-template control (designated as –RT) was included for each reaction. Additionally, cDNA from MMiPS, an iPSC line established from HDDPCs in our laboratory^11^, was used as a positive control. PCR primers are listed in Supplementary Table 1^11,25–27^. The products (5 µL) were analysed by 2% agarose gel electrophoresis and visualised after ethidium bromide staining.

### Gene microarray analysis

For the oligonucleotide-based microarray analysis, total RNA was extracted from cells using the RNA Mini Kit. Microarray analysis was performed with a 3D-Gene® Human Oligo chip 25k (Toray Industries Inc., Tokyo, Japan). For efficient hybridisation, this microarray adopted columnar structure to stabilize spot morphology and to enable micro bead agitation. Total RNA was labelled with Cy5 using the Amino Allyl MessageAMP II aRNA Amplification Kit (Applied Biosystems, CA, U.S.A.). The Cy5-labelled aRNA pools were mixed with hybridisation buffer and hybridised for 16 h. The hybridisation was performed according to the supplier’s protocols (www.3d-gene.com). The hybridisation signals were obtained using a 3D-Gene Scanner (Toray Industries Inc.) and processed by 3D-Gene Extraction software (Toray Industries Inc.). Detected signals for each gene were normalised using a global normalisation method described in the guide provided by the Toray Industries Inc. (the median of the detected signal intensity was adjusted to 25).

### *In vitro* differentiation induction assay

Primary HDDPCs and HDDPCs 13 days after transfection were seeded onto a gelatin-coated 6-well plate containing MEMα/20% FBS. After culturing to 80–90% confluency, the medium was changed to differentiation-inducing medium. To test ability hiTSC ability to differentiate into neurogenic and osteogenic cells, the method described in our previous study was used^24^.

To induce adipogenic differentiation, the cells were incubated in adipogenic differentiation medium (#05-330-1B, Biological Industries Ltd, Kibbutz Beit Haemek, Israel) for 14 days and stained with Sudan black B (#303 A, Daiichi Pure Chemicals Co., Ltd., Tokyo, Japan) to stain fats.

To induce EB formation, portions of HDDPC-iPSC colonies were dissected mechanically using a pipette tip under a stereomicroscope and then seeded onto an ultra-low attachment 96-well plate (#MS-9035X; Sumitomo Bakelite Co., Ltd., Tokyo, Japan) with DMEM/10% FBS. After 7 days of culture, emerging EBs were transferred onto gelatin-coated chamber slides (#154526JP, Iwaki Glass) and cultured for another 5 days in DMEM/10% FBS to allow enhanced differentiation into various cell types.

### *In vivo* teratoma formation assay

The potential of HDDPCs to form teratoma *in vivo* at 13 days after the 1st transfection with reprogramming factors was assessed using a recently established method termed ‘IPPCT’^12^, which allows growth and differentiation of a small number of iPSCs through grafting into the pancreatic parenchyma of nude mice by a glass injection needle under observation using a dissecting microscope.

Briefly, a solution (1–3 μL) containing HDDPCs (approximately 10^4^) suspended in MEMα/20% FBS was injected into the pancreatic parenchyma of anesthetised Balb/c-nu/nu mice. The injections were performed at a total of 3 different sites in each pancreas. In the positive control, iPSCs (approximately 10^4^) were also similarly injected. At 1.5 months after transplantation, the pancreas was inspected for the presence or absence of teratomas. When teratomas were detectable, they were dissected from the pancreas and fixed with 4% PFA at 4 °C for 4 days for preparing tissue sections for pathological analysis.

## Supplementary information

Supplementary Information

Supplemental Dataset
