# Supplementary material for: Repeated human deciduous tooth-derived dental pulp cell reprogramming factor transfection yields multipotent intermediate cells with enhanced iPS cell formation capability
Source: Sci Rep. 2019 Feb 6;9:1490. doi: 10.1038/s41598-018-37291-2 (PMC6365514; doi:10.1038/s41598-018-37291-2)
Supplement: Supplementary file 1 — Supplementary Information [file 41598_2018_37291_MOESM1_ESM.pdf]

Title: Repeated human deciduous tooth-derived dental pulp cell reprogramming factor transfection yields multipotent intermediate cells with enhanced iPS cell formation capability

Authors: Miki Soda[1], Issei Saitoh[1,*], Tomoya Murakami[1], Emi Inada[2], Yoko Iwase[1], Hirofumi Noguchi[3], Shinji Shibasaki[4], Mie Kurosawa[1], Tadashi Sawami[1], Miho Terunuma[5], Naoko Kubota[2], Yutaka Terao[6], Hayato Ohshima[7], Haruaki Hayasaki[1] & Masahiro Sato[8]

Affiliations: [1] Division of Pediatric Dentistry, Graduate School of Medical and Dental Science, Niigata University, Niigata, Japan.

[2] Department of Pediatric Dentistry, Kagoshima University Graduate School of Medical and Dental Sciences, Kagoshima, Japan.

[3] Department of Regenerative Medicine, Graduate School of Medicine, University of the Ryukyus, Okinawa, Japan.

[4] Faculty of Dentistry, Niigata University, Niigata, Japan.

[5] Department of Oral Biochemistry, Graduate School of Medical and Dental Sciences, Niigata University, Niigata, Japan.

[6] Division of Microbiology and Infectious Diseases, Graduate School of Medical and Dental Sciences, Niigata University, Niigata, Japan.

[7] Division of Anatomy and Biology of the Hard Tissue, Graduate School of Medical and Dental Sciences, Niigata University, Niigata, Japan.

[8] Section of Gene Expression Regulation, Frontier Science Research Center, Kagoshima University, Kagoshima, Japan.

*Corresponding Author: Issei Saitoh, D.D.S., Ph. D.
Division of Pediatric Dentistry
Graduate School of Medical and Dental Science, Niigata University
2-5274 Gakkocho-dori, Chuo-ku, Niigata 951-8514, Japan
Tel: +81 – 25 – 227 – 2911; Fax: +81 – 25 – 227 – 6911
E-mail: isaito@dent.niigata-u.ac.jp

Supplementary Figure Legends:

Figure S1: (i) Typical iPSC morphology of the HDDPC-derived iPSC colony derived from one of 12 lines generated after quadruple transfections. (ii), (iii) Morphology of the HDDPC-derived iPSCs derived from the other two of 12 lines generated after quadruple transfections. These two lines failed to show typical iPSC morphology. Bar = 500 µm.

Figure S2: RT-PCR analysis of mRNA expression for endogenous *OCT3/4* and *SOX2*. Lanes ① Primary HDDPCs, ② HDDPCs after the single transfection, ③ HDDPCs after the double transfection, ④ HDDPCs after the triple transfection, ⑤ no template control (designated as –RT), and ⑥ iPSCs established from HDDPCs in our laboratory[11] (used as positive control). PCR primers are listed in Supplementary Table 1. M, 100-bp ladder markers.

Figure S3: RT-PCR analysis of mRNA expression for endogenous *NANOG* and *KLF4*. Lanes ① Primary HDDPCs, ② HDDPCs after the single transfection, ③ HDDPCs after the double transfection, ④ HDDPCs after the triple transfection, ⑤ no template control (designated as –RT), and ⑥ iPSCs established from HDDPCs in our laboratory[11] (used as positive control). PCR primers are listed in Supplementary Table 1. M, 100-bp ladder markers.

Figure S4: RT-PCR analysis of mRNA expression for endogenous *TNSALP* and *GAPDH*. Lanes ① Primary HDDPCs, ② HDDPCs after the single transfection, ③ HDDPCs after the double transfection, ④ HDDPCs after the triple transfection, ⑤ no template control (designated as –RT), and ⑥ iPSCs established from HDDPCs in our laboratory[11] (used as positive control). PCR primers are listed in Supplementary Table 1. M, 100-bp ladder markers.

Supplementary Table Legend:

Table S1: Primer sets used for RT-PCR analysis.

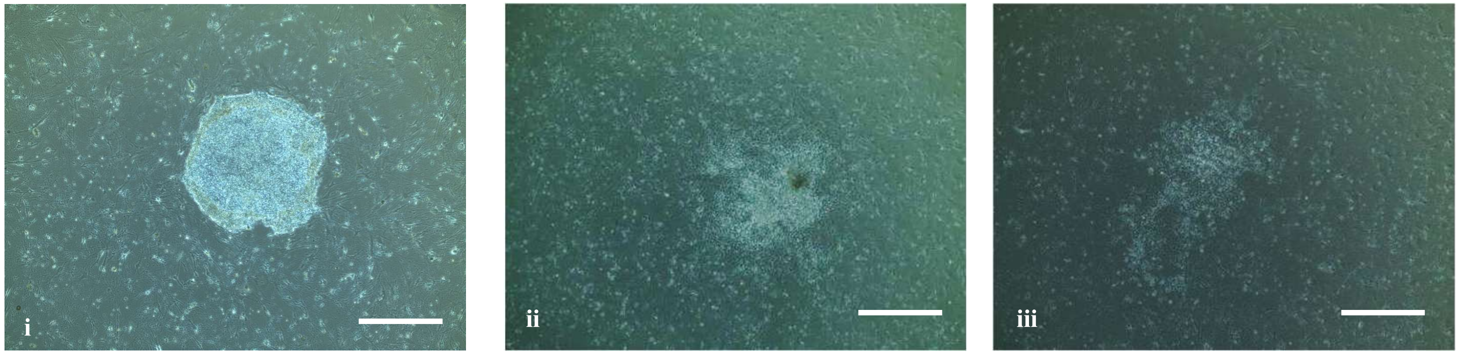

Figure S1

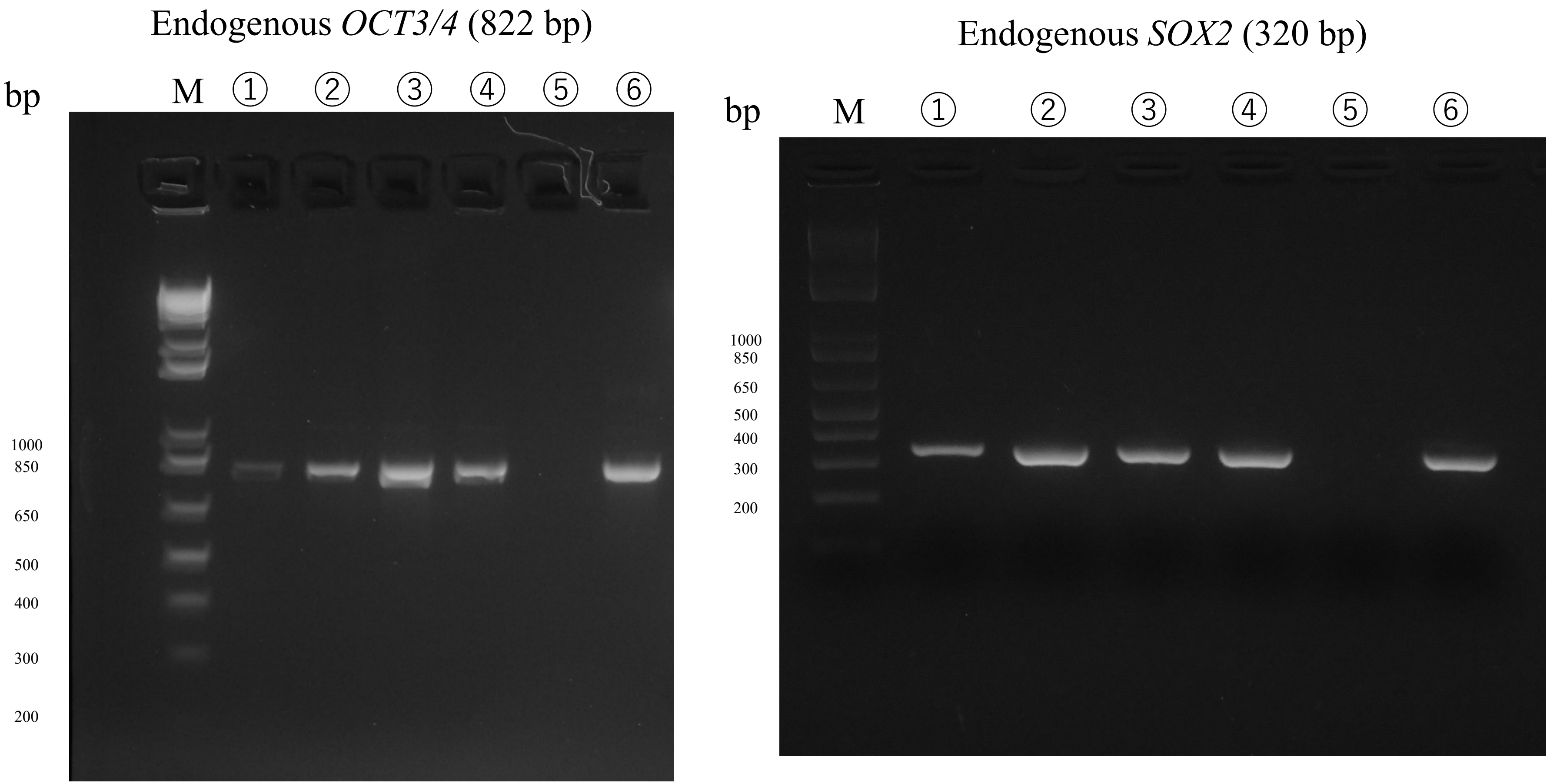
Endogenous OCT3/4 (822 bp)
bp
M
①
②
③
④
⑤
⑥
1000
850
650
500
400
300
200
Endogenous SOX2 (320 bp)
bp
M
①
②
③
④
⑤
⑥
1000
850
650
500
400
300
200

Figure S2

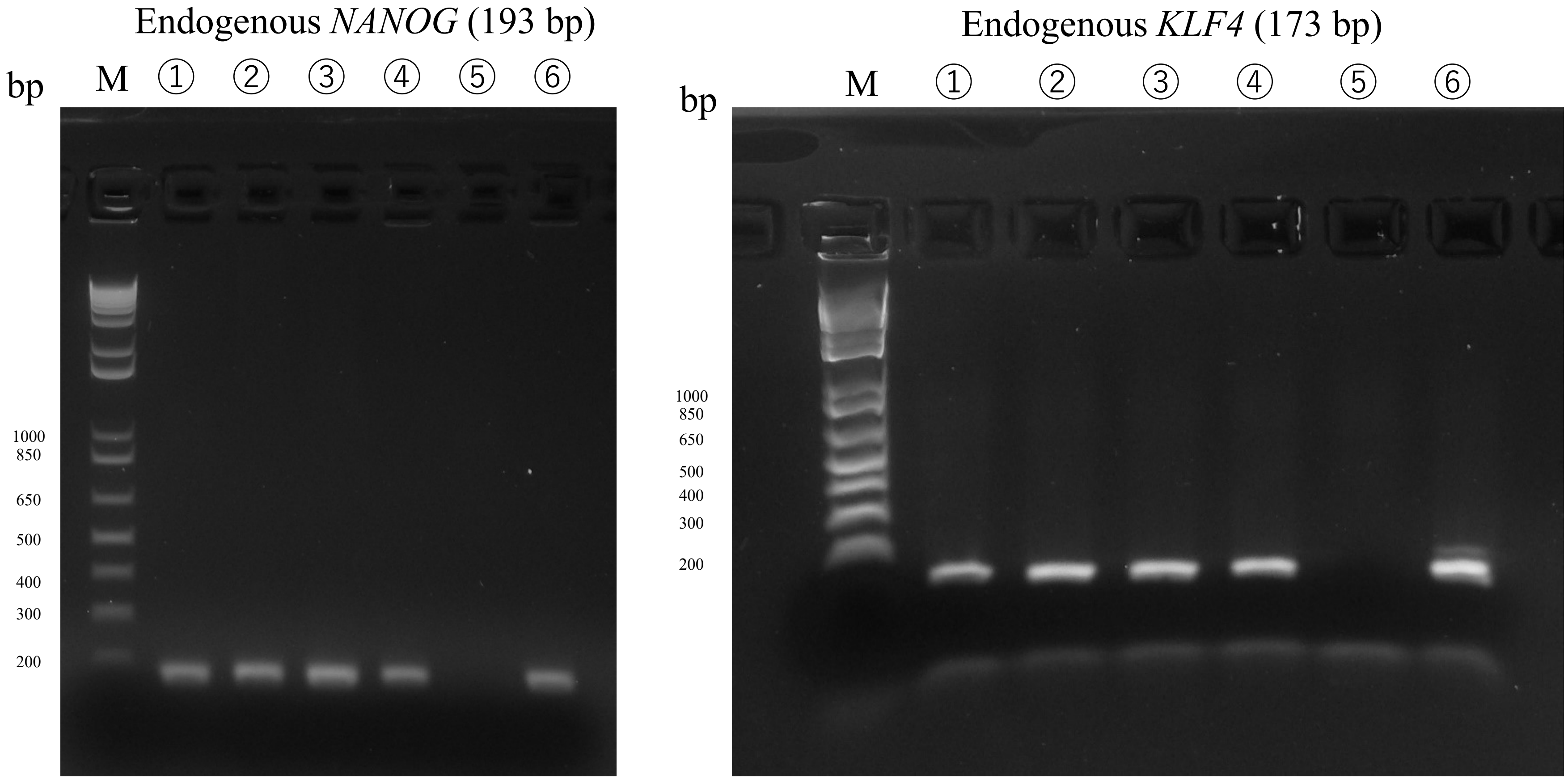

Endogenous *NANOG* (193 bp)
bp
M ① ② ③ ④ ⑤ ⑥
1000
850
650
500
400
300
200
Endogenous *KLF4* (173 bp)
bp
M ① ② ③ ④ ⑤ ⑥
1000
850
650
500
400
300
200

Figure S3

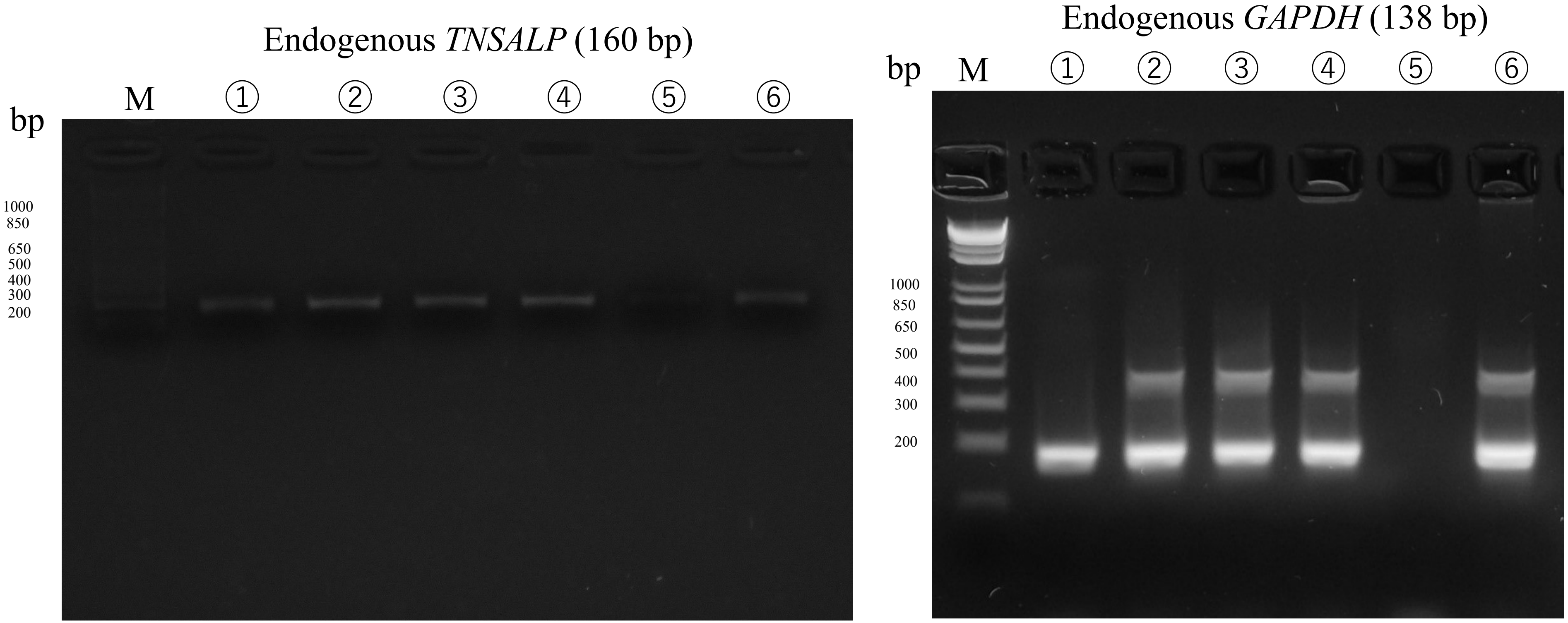
Endogenous *TNSALP* (160 bp)
bp
M
①
②
③
④
⑤
⑥
1000
850
650
500
400
300
200
Endogenous *GAPDH* (138 bp)
bp
M
①
②
③
④
⑤
⑥
1000
850
650
500
400
300
200

Figure S4

Table S1: Primer sets used for RT-PCR analysis

Gene	Forward Primer (5′-3′)	Reverse Primer (5′-3′)	Product Size (bp)	Reference
OCT3/4	ATTTCACCAGGCCCCCGGCT	GCTGATCTGCTGCAGTGTGGGT	822	Ref. 11
SOX2	AGGACCAGCTGGGCTACCCG	GGCGCCGGGGAGATACATGC	320	Ref. 11
NANOG	TTGGAAGCTGCTGGGGAAG	GATGGGAGGAGGGGAGAGGA	193	Ref. 27
KLF4	CGTGCTGAAGGCGTCGCTGA	GGGTGCACGAAGAGACCGCC	173	Ref. 28
TNSALP	TGGCCCCCATGCTGAGTGACAC	TGGCGCAGGGGCACAGCAGAC	160	Ref. 29
GAPDH	GCACCGTCAAGGCTGAGAAC	TGGTGAAGACGCCAGTGGA	138	Ref. 27
